# Using Linkage Analysis to Detect Gene-Gene Interaction by Stratifying Family Data on Known Disease, or Disease-Associated, Alleles

**DOI:** 10.1371/journal.pone.0093398

**Published:** 2014-04-01

**Authors:** Barbara Corso, David A. Greenberg

**Affiliations:** 1 National Council Research, Neuroscience Institute, Padova, Italy; 2 Battelle Center for Mathematical Medicine, Nationwide Children's Hospital, Columbus, Ohio, United States of America; 3 Department of Pediatrics, Wexner Medical Center, Ohio State University, Columbus, Ohio, United States of America; Oklahoma Medical Research Foundation, United States of America

## Abstract

Detecting gene-gene interaction in complex diseases is a major challenge for common disease genetics. Most interaction detection approaches use disease-marker associations and such methods have low power and unknown reliability in real data. We developed and tested a powerful linkage-analysis-based gene-gene interaction detection strategy based on conditioning the family data on a known disease-causing allele or disease-associated marker allele. We computer-generated multipoint linkage data for a disease caused by two epistatically interacting loci (A and B). We examined several two-locus epistatic inheritance models: dominant-dominant, dominant-recessive, recessive-dominant, recessive-recessive. At one of the loci (A), there was a known disease-related allele. We stratified the family data on the presence of this allele, eliminating family members who were without it. This elimination step has the effect of raising the “penetrance” at the second locus (B). We then calculated the lod score at the second locus (B) and compared the pre- and post-stratification lod scores at B. A positive difference indicated interaction. We also examined if it was possible to detect interaction with locus B based on a disease-marker association (instead of an identified disease allele) at locus A. We also tested whether the presence of genetic heterogeneity would generate false positive evidence of interaction. The power to detect interaction for a known disease allele was 60–90%. The probability of false positives, based on heterogeneity, was low. Decreasing linkage disequilibrium between the disease and marker at locus A decreased the likelihood of detecting interaction. The allele frequency of the associated marker made little difference to the power.

## Introduction

It is clear that the expression of common disease depends on the interaction of multiple loci. Over the past decade, the technique of choice for identifying disease loci has been association analysis, particularly Genome Wide Association Studies (GWAS). These studies, which usually involve thousands, if not tens of thousands of subjects, assume that finding highly significant statistical differences between marker allele frequencies in case and control populations would guarantee that a gene strongly influencing disease expression would be discovered. However, the disease relative risk seen for most of these associated genes was so low as to call into question the utility of further pursuing these genes. Furthermore, although gene-gene interaction is viewed as critical for understanding common disease expression, the statistical tests used to detect gene-gene interaction from association data are weak.

There are several current techniques to detect interaction from association data. One chooses two known (or hypothesized) alleles or genotypes at two loci and applies a regression model that includes main effects and interaction terms and then tests if the interaction terms are zero. There are variations that use logistical regression or multinomial regression on case-control data, case-only data, or family-based association data. One could conceivably do a pair-wise test of all SNPs in a GWAS to search for an association but achieving a result with enough statistical significance to survive a correction for the number of tests would be difficult. The rational alternative is to choose loci that show association by themselves and test for interaction. There are also data-mining approaches and methods that look for all combinations of alleles that appear to influence disease expression [1].

However, there are disadvantages to all these approaches.

1. The power to detect association is reduced in the face of allelic heterogeneity. Thus, the existence of multiple alleles at a locus with differing influences on disease, affect association detection. If one adds to that varying amounts of linkage disequilibrium between disease allele and marker SNP [2] and differing interactions with alleles at other loci, interaction detection becomes more difficult.
2. Some approaches would require an enormous sample size and overwhelming statistical significance to correct for the number of tests [1].
3. The biological significance of a positive result is not immediately clear. Given that most GWAS-discovered associations have low relative risks, it is unclear how much more evidence for risk could be detected by looking for interaction. If an exhaustive pair-wise search approach is used, is unclear what detecting an interaction involving two alleles at different loci would mean in the absence of evidence of association between the disease and each allele independently.

Combined with the low relative risks, the association analysis-based approach to detecting gene-gene interaction has disadvantages. Given the enormous investment necessary to determine if there is a biological basis for the statistical observation, a more robust approach that is more likely to ensure biological significance is needed.

Linkage analysis identifies gene locations based on family data. It has the advantage of using inheritance information rather than gene frequency differences between case versus control populations. Linkage analysis can best detect loci that have a major effect on disease expression, a characteristic that can be viewed as either an advantage or a disadvantage, but, given the state of our knowledge about common disease, we consider this as a distinct advantage.

We recently showed how linkage analysis could be used to both prove the existence of gene-gene interaction and uncover additional loci that contribute to disease [3]. We successfully showed evidence for a second locus that interacts with *BMPR2* locus mutations to contribute to the expression of Familial Pulmonary Arterial Hypertension (FPAH). The *BMPR2* mutations, although unequivocally involved in FPAH expression, have a low penetrance, an observation that, in the absence of any identifiable environmental influence, can only be explained by gene-gene interaction [3].

Our approach to detecting interaction stratifies the linkage data on alleles at a locus known to cause, or be associated with, the disease. In this work, we use extensive computer simulation to explore the statistical characteristics of using linkage stratification to learn the strengths and the limitations, power and efficiency of the method so that it can be a useful and a well-understood tool to identify gene-gene interaction.

The linkage-based method we present appears quite robust. Linkage is insensitive to allelic heterogeneity, so the presence of multiple alleles at a locus does not affect power. The finding of a causative or associated allele under a linkage peak can be used to enhance the detection of other linked loci that interact with that allele.

## Methods

### Epistatic Genetic Models

In an epistatically interacting two-locus disease inheritance model, both loci are necessary for disease expression, i.e., the disease genotype at both loci must be present in order for a subject to be affected. Depending on the gene frequencies of the disease alleles at the two loci, the “apparent” penetrance of the disease will vary, viewed as a single locus model based on either one of the loci alone, as will the expected proportion of affected siblings (the ascertainment-corrected segregation ratio) [4]. What has traditionally been interpreted as “reduced penetrance” is based on the assumption of a single-locus model. In a two-locus epistatic model, the so-called reduced penetrance at a locus, which is a characteristic ascribed to the disease when assuming a single-locus model of inheritance, is caused by the second locus, even though the penetrance of the disease genotype, which by definition includes both loci, is full. The basis of our approach is, in effect, to make the model a one-locus model by ensuring almost everyone in the dataset has the disease genotype at the first (known) locus. Then, the presence of the disease alleles at the other locus will determine affectedness status, creating a single-locus model.

The model we studied is an epistatic two-locus system requiring disease genotypes at both loci. At one locus (locus A), a known disease-causing allele is segregating. We first linkage analyze the data at locus B (the test locus) assuming the correct parameters, including penetrance (the reduced penetrance being caused by the effect of locus A), and record the lod score. We then stratify the data by including only carriers of the disease allele or genotype at the known locus A. Then, we perform a second linkage analysis at locus B (which, in an analysis using real data, might include all markers in the rest of the genome). By stratifying the data so that subjects without the disease allele at locus A are excluded, everyone remaining (except necessary connecting subjects) is a carrier of the disease allele or genotype at A. Thus, we effectively raise the penetrance at the second, interacting locus (B) because non-carriers of the disease allele at A cannot be affected and, by eliminating them, we are excluding “non-penetrant” carriers of the disease allele at locus B. Thus, after stratification, all subjects will have the disease genotype at locus A (except perhaps parents) and only those who are affected will have the disease genotype at B.

The nature of the interaction we studied was always a two-locus epistatic model, in which both loci together are causative. However, we also examined the case where the allele leading to exclusion of subjects was not the causative allele at locus A but an allele in linkage disequilibrium (LD) with the causative allele and thus was associated with the disease (see below). In that case, also, we include in the stratified (or pruned) analysis only those offspring carrying the associated allele and who are thus more likely to carry the disease allele at a known locus (and their parents) by dint of that association.

The test statistic we devised compared the evidence for linkage at B after pruning with the evidence for linkage at B using the unpruned data. We tested some variation from the above ideal epistatic condition that may model other realistic situations.

### Simulation parameters

**Figure 1:**
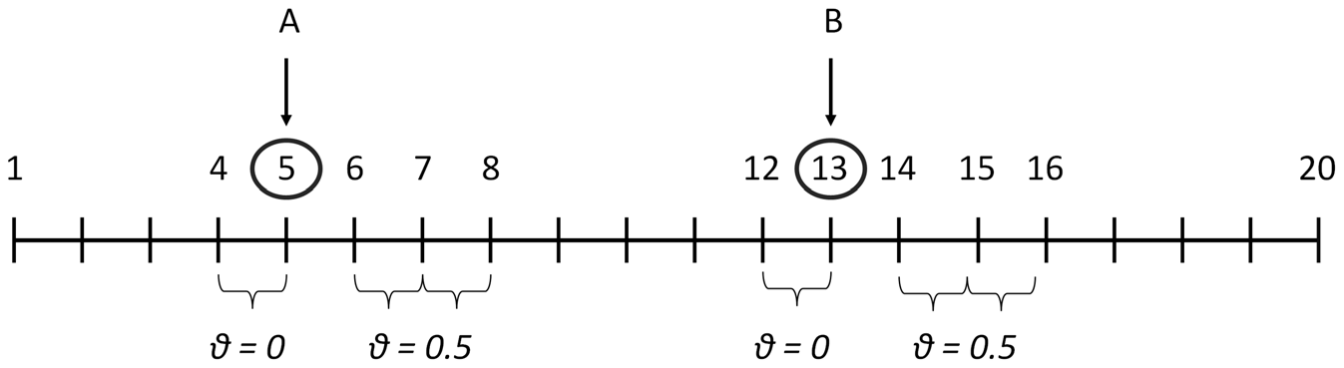
Simulation map. All recombination fractions, except those labeled, were set to 0.001.

We generated multipoint linkage data for a disease caused by two unlinked, epistatically-interacting loci (A and B) using a modification of the program Caleb (http://potato.nationwidechildrens.org/caleb) [5] called Xcaleb. Caleb generates multipoint family data in which two disease loci interact to produce the disease and for which the details of the interaction can be specified. In addition to the two disease loci, the program lets the user specify up to 18 single nucleotide polymorphism (SNP) marker loci of arbitrary gene frequency and also allows specification of pair-wise linkage disequilibrium (LD) between loci. In all our simulations, we fixed the first disease locus (locus A) at position 5 and the second disease locus (locus B) at position 13 on the simulated “chromosome” (see Figure 1). These loci were not linked to each other (recombination fraction (θ)  = 0.5). We then calculated the multipoint lod scores and specifically focused on the lod score at the test locus B.

The genetic distances between loci (θ) used to generate the linkage data are shown in Figure 1. Some of the loci between disease locus A and disease locus B were separated by recombination fractions that ensured A and B were unlinked. Recombination fractions between the other markers surrounding the disease loci were fixed at 0.001.

The LD measure, D', between the disease allele at locus A and marker allele 1 at locus 4 was set to 1. The gene frequency of the disease allele matched that of allele 1 (e.g., r^2^ = 1); thus, allele 1 at the marker always occurred together with the disease allele at locus A and never with the normal allele. The recombination fraction between locus A and the locus 4 marker was set to 0 (see Figure 1). There was no LD, and thus no disease-marker association, between other marker alleles.

Each experiment consisted of 500 datasets of 50 families each. The ascertainment criteria were set as follows: all simulated families were two-generation pedigrees and were required to have *at least* 1 affected offspring and a minimum of 4 members in the pedigree (i.e., a two-child nuclear family). The maximum family size consisted of two parents and 10 offspring. The family size distribution was based on Cavalli-Sforza and Bodmer [6].

We tested four different fully penetrant epistatic two-locus disease models (“fully penetrant” meaning that the disease genotype at both loci must be present for disease expression). The models were: a dominant-dominant model (DD), a dominant-recessive model (DR), a recessive-dominant model (RD) and a recessive-recessive model (RR) [4], [7]. The disease-causing genotypes are (capital letters designate disease alleles):

DD model: AABB, AABb, AaBB, AaBb;

DR model: AABB, AaBB;

RD model: AABB, AABb;

RR model: AABB.

When the disease model at a locus was dominant, the disease allele frequency was fixed at 0.1; when the model was recessive, the frequency was set to 0.2. The marker loci allele frequencies were all set to 0.5.

### Pruning/Stratification

Each simulated family was analyzed twice. In the first analysis, evidence for linkage at locus B was calculated using all families and family members. We refer to this as the “unstratified” analysis. For the second analysis only the offspring carrying the disease allele at locus A were included. (Parents were also included, irrespective of their genotype.) We refer to this as the “stratified” (or “pruned”) analysis.

**Figure 2:**
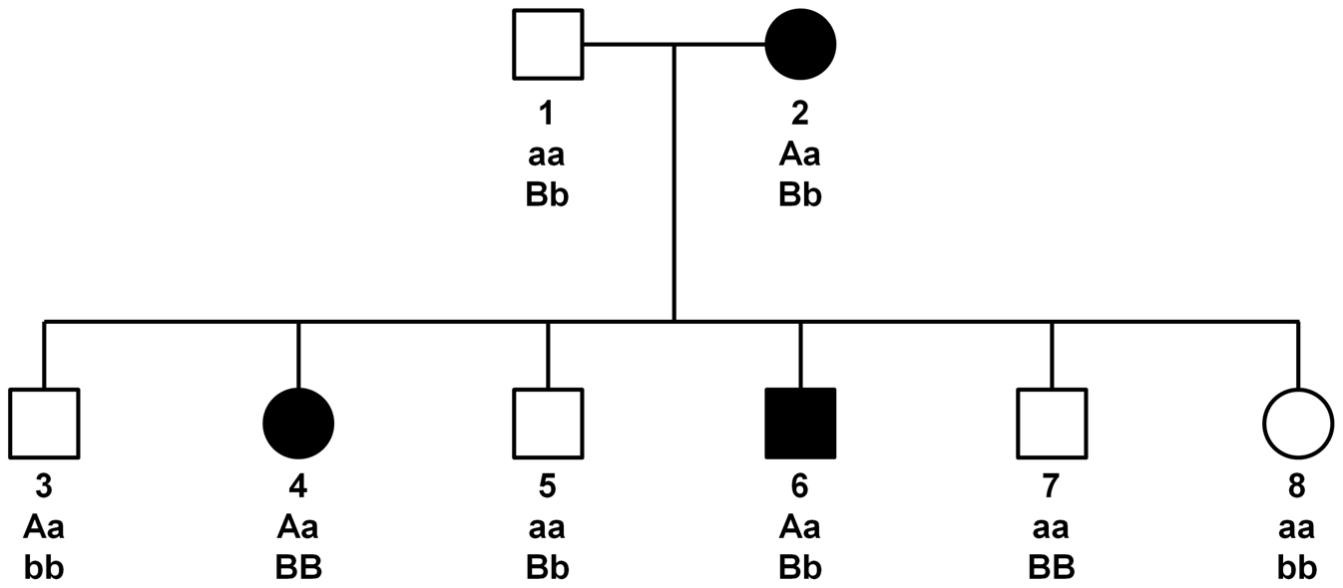
Example of simulated family under a DD model. Capital letters designate disease alleles.

We illustrate the system with a example using the DD model:

Figure 2 shows one of the simulated families when a DD model was specified. The letters under each pedigree member are the genotypes at the two epistatic loci. (Capital letters designate disease alleles.) There are three affected subjects: the mother (id 2) and two children (id 4 and id 6). In the first analysis, all subjects were included in the linkage computation. In the second analysis we eliminated offspring 5, 7 and 8, and re-calculated the lod score.

This procedure means that there were fewer subjects in the “stratified” analysis than in the “unstratified” analysis. Also, the number of families in the “stratified” analysis could be lower than the number of families in the “unstratified” analysis because removal of non-carrier offspring can turn families with two offspring into uninformative trios.

### Analysis parameters

The correct (generating) map (see Figure 1) was used in the analysis. The analysis penetrance was set to 0.5 for each analyzed model [8]. The GENEHUNTER program [9], [10], was used to compute the multipoint lod scores.

In the analysis, the assumed disease allele frequencies and mode of inheritance were always matched to the generating values for locus B.

### Test statistic

In order to devise a test to determine if we can detect interaction between loci A and B and define its reliability, we compared the lod score at locus B calculated under the unstratified analysis to the one obtained after pruning (recall, pruning occurs at locus A). For both unstratified and stratified analyses, we calculated the maximum lod score in the linkage interval containing locus B for each dataset. We then calculated the lod score difference between the stratified and unstratified analysis for each dataset. We also determined the means over all data sets for a given model.

The test statistic is based on these differences and can be written as
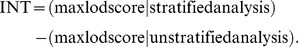



The INT statistic can be interpreted as follows:

- If the stratified and the unstratified lod scores are both positive:

A *positive INT* occurs when the lod score at locus B is found to be higher after pruning (i.e. when non-carriers of the disease allele at locus A are removed) and indicates evidence of interaction. The lod score can increase only if locus A and B interact (which we show below when we observe how the INT behaves when heterogeneity, rather than epistasis, is simulated). By including only carriers of the disease allele at locus A, we are effectively raising the penetrance at locus B by eliminating those subjects who do not have the disease genotype at locus A and therefore cannot be affected with the disease if it arises from an interaction between A and B. If a *negative or null INT* is obtained, it suggests that there is no interaction between the loci because the apparent penetrance at locus B remains the same or decreases, indicating that eliminating non-disease genotypes at locus A has no effect on the linkage signal (or a negative effect) at locus B.

- Similarly, if the unstratified lod score is negative and the stratified lod score is positive:

This would mean that conditioning on carriers of the disease genotype at locus A increases the evidence for linkage at locus B. Thus, raising the penetrance at locus B (by eliminating family members incapables of being affected because they do not have the disease genotype at locus A, but who have the disease genotype at locus B) increases locus B's detectability. This happened in Rodriguez-Murillo et al. [3].

- If the unstratified lod score is positive and the stratified lod score is negative:

This would yield only *negative values* for the INT statistics, indicating that conditioning on carriers of the locus A disease allele gives us evidence against linkage while the unstratified analysis produces evidence in favor of linkage. This suggests that the stratification process has eliminated evidence in favor of linkage at locus B, suggesting there is no relationship between the loci.

### Heterogeneity

A linkage analysis can produce evidence of multiple loci. These signals, if not occurring by chance, can be due either to epistatically interacting loci or to loci that independently produce the phenotype, i.e., genetic heterogeneity. To test whether the mere existence of multiple, but non-interacting loci could lead to false indications of interaction, we tested stratification when the disease phenotype is independently caused by two different loci. For this scenario, we also simulated and analyzed heterogeneity models analogous to the epistatic models, designated: D+D, D+R, R+D and R+R with the same parameters as described above. The simulated and analysis penetrances for the two independent loci were both set to 0.5.

### Associated allele

Above, we illustrated the situation in which there is a known disease allele at locus A, i.e., the ideal case. We now ask: can the existence of a disease-marker *association*, rather than an actual disease-causing mutation at locus A, be used to identify interaction? We explored how strong the association between the disease allele and the associated marker allele needs to be in order to demonstrate interaction between the loci using the stratification approach. We tested three scenarios:

1. We observed the effect of changing the strength of the association between the disease-associated allele (at locus A) and the marker allele at marker locus 4 (see Figure 1) by changing the LD between those two alleles. We varied the LD, examining the D' values: 0, 0.2, 0.4, 0.6, 0.8 and 0.9.
2. We tested the situation in which the D' is fixed at a value of 1 but the allele frequency of the associated marker allele is varied. We tested allele frequencies of 0.1, 0.2, 0.3, 0.4 and 0.5.
3. We varied both D' and the associated marker allele frequency simultaneously.

The above disease-marker association scenarios weaken the association between the associated allele and the locus A disease allele compared to the first analysis in which the LD between the marker and the disease allele was unity. Consequently, the presence of the associated allele would not always signal the presence of the disease allele at locus A. Accordingly, when the data are stratified, the evidence for interaction would be weakened because in some carriers of the locus 4 marker, that allele would not be syntenic with the disease allele at A. Thus, the non-carriers of disease locus at A would be less likely to be eliminated.

## Results

### 1. Pruning based on the presence of the disease allele

#### a) Effect of pruning on sample size

When the inheritance model at locus A is dominant, the reduction in the number of families was approximately 27% (29% for the DD model and 24% for the DR model) and the reduction in the number of subjects was approximately 18% (20% for DD model and 16% for DR model). When locus A was recessive, the number of families after stratification was only 53% of the number in the unstratified analysis (55% for the RD model and 51% for the RR model). The number of subjects reduced to about 37% of the original number when locus A was recessive (34% for RD model and 39% for RR model) (Table 1).

**Table 1:** Results from multipoint linkage simulation.

EPISTASIS								
	DD		DR		RD		RR	
	Unstr	Str	Unstr	Str	Unstr	Str	Unstr	Str
*a. Family structure a.*								
	25 000	17 690	25 000	18 944	25 000	13 869	25 000	12 669
# Subjects	107 825	85 895	112 576	94 408	97 714	64 321	94 665	57 672
*b. Mean Lod scores (*±*sd)*								
Overall	5.5±2.0	6.4±1.7	7.6±2.4	8.6±2.1	1.9±1.7	3.1±1.2	3.0±2.0	4.6±1.6
if unstr Lod score ≥0	5.5±2.0	6.4±1.7	7.6±2.4	8.6±2.1	2.3±1.4	3.4±1.0	3.3±1.8	4.8±1.5
if unstr Lod score ≥1.5	5.6±1.9	6.5±1.6	7.6±2.4	8.6±2.1	3.0±1.1	3.7±1.0	3.7±1.5	5.1±1.4
if unstr Lod score <0	-	-	-	-	−0.7±0.6	1.8±0.7	−0.9±0.7	2.4±1.2
*c. Percent of Datasets (of 500) with*								
Lod score <0	0	0	0	0	14.2%	0.2%	7.0%	0.2%
Lod score ≥0	100%	100%	100%	100%	85.8%	99.8%	93.0%	99.8%
Lod score ≥1.5	97.2%	99.8%	100%	100%	58.4%	93.2%	78.6%	97.4%
Lod score ≥3	88.8%	98.4%	97.8%	100%	26.4%	54.0%	49.6%	85.6%

HETEROGENEITY								
	D+D		D+R		R+D		R+R	
	Unstr	Str	Unstr	Str	Unstr	Str	Unstr	Str
*a. Family structure*								
# Families	5 000	2 086	5 000	3 109	5 000	587	5 000	1 322
# Subjects	28 124	10 138	28 557	15 183	28 310	2 661	29 069	6 052
*b. Mean Lod scores (*±*sd)*								
Overall	−0.6±1.9	−1.6±1.3	−8.2±3.2	−6.4±2.6	2.5±2.0	−0.4±0.6	−1.4±3.2	−2.6±1.8
if unstr Lod score ≥0	1.5±0.9	−0.5±0.8	-	-	2.9±1.6	−0.3±0.6	1.8±1.3	−1.4±1.1
if unstr Lod score ≥1.5	2.2±0.6	−0.2±0.9	-	-	3.4±1.3	−0.3±0.5	2.9±1.0	−1.1±0.7
if unstr Lod score <0	−1.8±1.3	−2.2±1.1	−8.2±3.2	−6.4±2.6	−1.2±0.8	−0.8±0.8	−3.1±2.6	−3.2±1.7
*c. Percent of Datasets (of 100) with*								
Lod score <0	65%	88%	100%	100%	11%	67%	64%	94%
Lod score ≥0	35%	12%	0	0	89%	33%	36%	6%
Lod score ≥1.5	17%	0	0	0	71%	0	17%	0
Lod score ≥3	3%	0	0	0	39%	0	9%	0

Epistasis: disease is caused by two unlinked interacting loci; Heterogeneity; disease is caused by two independent loci. For each unstratified (Unstr) and stratified (Str) analysis and each model (DD/D+D, DR/D+R, RD/R+D and RR/R+R), the table contains: a) Family structure: number of families and subjects; b) Lod scores: mean (±sd) that satisfy selected row's condition; c) Percentages of datasets that satisfy selected row's condition.

When larger datasets with fewer replicates were simulated (100 dataset/100 families), we observed the same percentage reduction in both the numbers of families and subjects (data not shown).

#### b) Effect of pruning on lod scores

**Figure 3:**
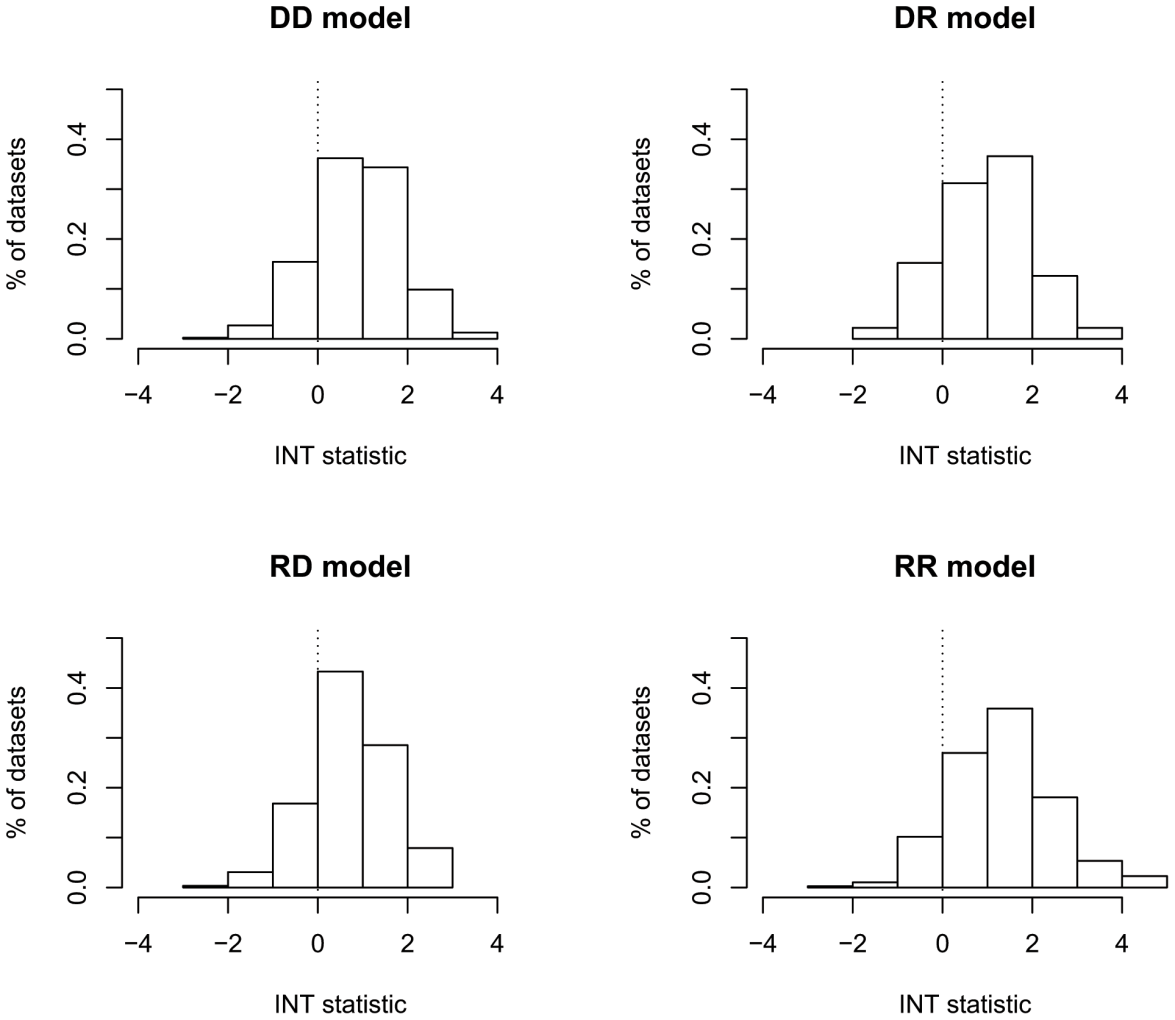
Distribution of INT statistic when unstratified lod score was at least 1.5 units. Vertical dotted lines emphasize the boundary between negative and positive INT values.

Although stratification decreases dataset size both in families and subjects, if interaction exists, the lod scores generally increase after stratification, making the INT positive. Figure 3 shows the distribution of the INT for the four inheritance models when the unstratified lod score reached a value of at least 1.5. We chose a cutoff of 1.5 after we observed that the average increase in lod score with pruning was about 1.0. Thus, after pruning, the lod score for the dataset would, on the average, be 2.5 and thus usually be highly suggestive of linkage at a genome-wide level [11]. In almost 80% of the cases, the lod score at locus B increased after pruning (i.e., INT>0) (Table 2).

**Table 2:** INT statistics under selected unstratified Lod scores values.

EPISTASIS								
	DD		DR		RD		RR	
	% pos INT	Mean INT	% pos INT	Mean INT	% pos INT	Mean INT	% pos INT	Mean INT
Unstr Lod score ≥0	82.2%	0.9±1.0	82.6%	1.0±1.0	86.2%	1.1±1.0	90.3%	1.5±1.2
Unstr Lod score ≥1.5	81.7%	0.9±1.0	82.8%	1.0±1.0	79.8%	0.7±0.9	88.5%	1.3±1.1
1.5 ≤ Unstr Lod score <3	97.6%	1.8±0.8	100%	2.2±0.8	90.0%	1.0±0.8	97.2%	1.9±1.0
Unstr Lod score <0	-	-	-	-	100%	2.4±0.8	100%	3.3±1.1

HETEROGENEITY								
	D+D		D+R		R+D		R+R	
	% pos INT	Mean INT	% pos INT	Mean INT	% pos INT	Mean INT	% pos INT	Mean INT
Unstr Lod score ≥0	2.9%	−1.9±0.9	-	-	3.4%	−3.2±1.5	2.8%	−3.2±1.4
Unstr Lod score ≥1.5	0	−2.4±0.8	-	-	0	−3.7±1.2	0	−4.0±0.8
1.5 ≤ Unstr Lod score <3	0	−2.4±0.9	-	-	0	−2.8±0.6	0	−3.4±0.5

For each situation (Epistasis and Heterogeneity) and each model (DD/D+D, DR/D+R, RD/R+D and RR/R+R) the percentage of positive INT statistics (% pos INT) are shown. Also shown are the corresponding INT means (±sd) when the unstratified (Unstr) Lod scores satisfy the selected row's condition. LD between marker 4 and disease locus A was set to 1 and marker 4 allele frequency was matched to disease locus A allele frequency.

#### c) INT statistic

The INT statistic was positive in almost 82% of the cases when locus A was dominant and 84% when it was recessive (when the unstratified lod score was equal or greater than 1.5). The most power to detect interaction was obtained under the RR model: 89% of the datasets produced a positive INT statistic.

The mean increases in the lod score for the different models after pruning when the unstratified lod score at locus B was greater or equal than 1.5 units were: DD: 0.9; DR: 1.0; RD: 0.7; RR: 1.3.

#### d) Choosing data sets based on the unstratified lod score

If we consider datasets that showed any positive value of the unstratified lod score at locus B (as opposed to only considering unstratified lod score values ≥1.5), the mean increases in the lod scores after pruning were: DD: 0.9; DR: 1.0; RD: 1.1; RR: 1.5. (Table 2). We emphasize that this increase in information for linkage occurred despite the stratification-caused decrease in both the number of families included in the analysis and in the total number of family members.

#### e) Increased linkage evidence using pruning

If we look only at datasets that had an unstratified lod score between 1.5 and 3 at locus B (i.e., positive but not statistically significant linkage evidence), whether the lod score rose to statistical significance depended on the underlying model. For the DD model, 95% of such datasets achieved a lod score greater than 3 after stratification. The percentages for the others models were: 100% of the DR datasets, 65% of the RD datasets and 87% of the RR datasets. Thus, pruning can also aid in confirming linkage in cases where the lod score is positive but not statistically significant (data not shown).

Pruning also could reveal evidence in favor of linkage at locus B that could not be seen otherwise. 71 (14%) of the RD datasets and 35 (7%) of the RR datasets showed a negative lod score at locus B under the unstratified analysis. After stratification, only 1 dataset (for both models) continued to show a negative lod score, while the mean lod score was 1.8 for the RD model and 2.4 for the RR model. Furthermore most of those datasets showing evidence against linkage at locus B increased the lod score to at least 1.5 after stratification (45 of the RD datasets (out of the 71 showing negative lod scores) and 25 (out of 35) of the RR datasets). For the DD and DR models, no dataset produced a negative unstratified lod score (Table 1).

### 2. There was no false evidence for interaction under heterogeneity

We tested whether there could be evidence for interaction (a positive INT) when interaction is not present, i.e., when the same phenotype can be caused independently by two different loci (genetic heterogeneity).

The reduction in the number of families and the number of subjects after stratification was much greater under heterogeneity than under epistasis. The number of families declined by 52%–88% and the number of subjects by about 47%–90%, with decreases in sample size when locus A was recessively inherited more pronounced than under dominant models (Table 1).

Under heterogeneity, a positive unstratified lod score might be anticipated (the loci are, in fact, linked to markers), but the presence of heterogeneous data will diminish the lod score. (These calculations did not use the heterogeneity lod score (HLOD).) When we looked at datasets under the D+R model, the lod score at locus B (the recessive locus under this model), was never positive before stratification and never became positive when stratified. Under the other models, 3% of the datasets had positive INT values (Table 2). When the unstratified D+D model lod score at locus B was positive, the mean unstratified locus B lod score was 1.5. After stratification that value decreased to -0.5. For the R+D model, the mean locus B lod score decreased from 2.9 to -0.3 after stratification and for the R+R model from 1.8 to -1.4. When the unstratified lod score at locus B was greater than 1.5, the INT statistic was *never* positive when heterogeneity existed (Table 2).

Thus, when heterogeneity exists, not only does stratification not increase the evidence for linkage at locus B, as it does when there is interaction, it causes a loss of linkage evidence.

### 3. Interaction with an associated marker allele rather than the locus A disease allele

#### a) The effect of D' between the associated allele and the locus A disease allele

We examined how the strength of association affects the INT statistic by varying the value of D' between the associated marker 4 allele and the disease allele at locus A.

We tested D' values of 0, 0.2, 0.4, 0.6, 0.8 and 0.9. In all these simulations, the associated allele frequency was matched to the locus A disease allele frequencies (0.1 for dominant and 0.2 for recessive).

**Figure 4:**
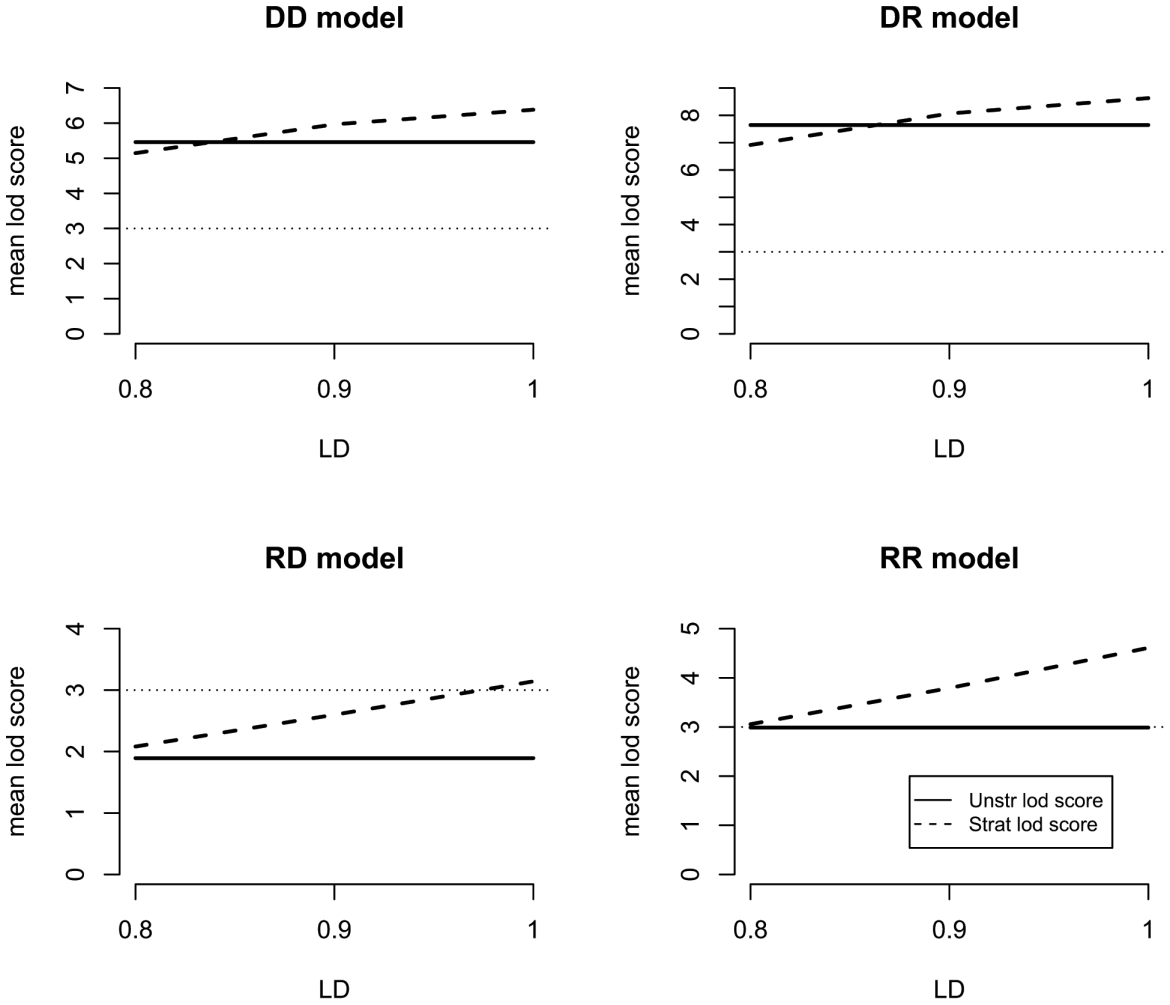
Lod score values varying the D' value between marker 4 and disease locus A. Unstratified lod score is represented by solid line and stratified lod score by dashed line. Horizontal dotted line identifies the threshold for significant evidence of linkage.

Figure 4 shows each model's mean lod score values for the unstratified analysis (solid line) and the stratified analysis (dashed line) at locus B, plotted against the D' values. When D'<0.8, there was no increase in the lod score after stratification for the RR and RD models. There was no increase for the DD and DR models, when D' is less than about 0.85.

Table 3 shows the percentage of datasets in which the INT statistic was positive when the unstratified lod score was positive. For the RD and RR models, a D' of at least 0.8 is needed in order to have 50% power to detect interaction (INT >0). For the DD and DR models the D' value needed to be at least 0.9 to reach 50% power.

**Table 3:** Percentage of datasets in which INT statistic is positive when unstratified lod score was positive.

	Model			
D' value	DD	DR	RD	RR
**0.8**	41.60	31.20	50.58	49.68
**0.9**	58.00	53.00	57.83	67.05
**1**	82.20	82.60	86.25	90.32

The findings when D' is unity (i.e., the associated allele is the disease allele at locus A) (part 2, above) showed there is greater than 80% power to detect gene-gene interaction. As D' decreases, the power drops precipitously. With a D' of 0.9, the power is 59% if the unstratified lod score is positive (Table 3).

Similar to the lack of any false positives when heterogeneity, rather than epistasis, existed, here were also no false positives under any D' values, although much more testing is necessary to arrive at a reliable type 1 error probability. We conclude that testing for gene-gene interaction using the proposed stratification procedure is unlikely to produce a false positive indication of gene-gene interaction.

#### b) The effect of varying the associated marker allele frequency

We also explored the effect on lod score varying the associated allele gene frequency together with D'. We tested marker allele frequencies of 0.1, 0.2, 0.3, 0.4 and 0.5. (Recall the associated allele frequency was 0.1 when locus A was dominant and was 0.2 when locus A was recessive.)

We found little dependence of the INT on the allele frequency at D' values of 0.8–1.0 (data not shown).

## Discussion

We have demonstrated that stratification is a useful strategy to detect epistatic gene-gene interaction in complex disorders. It is necessary to have identified a disease-causing or a disease-associated allele at a locus linked to the disease in order to test a possible interaction of that locus with another. While many disease-causing or -associated alleles are known, to our knowledge, there has been no linkage-based method to test for interactions of that known disease locus with other loci. In the current work, we have thoroughly explored the behavior of the stratification approach when applied to certain epistatic inheritance models. We have shown through extensive computer simulation tests that conditioning on a known disease-causing or disease-associated allele can demonstrate the existence of gene-gene interaction or confirm the existence of a second epistatically-interacting locus.

The proposed INT statistic indicated evidence of interaction up to 89% of the time (considering a starting lod score of 1.5 units) when interaction did exist, depending on the modes of inheritance of the two loci, among other factors. We anticipated that a major confounder in applying this method, as it is in all genetic analysis, was likely to be heterogeneity because multiple loci could be detected using linkage but for which no interaction would exist. We used the existence of heterogeneity as a basis for estimating the frequency of false positive evidence of interaction. To our surprise, we could detect essentially no false positives under heterogeneity. Nonetheless, heterogeneity is likely to be a confounder in real data because a locus that independently causes disease could mask the signal for two epistatically-interacting loci. However, the HLOD score allows us to detect loci even in the presence of heterogeneity [12]. The utility of the HLOD for the stratification approach to detecting interaction in the presence of heterogeneity is a question we are now investigating.

We estimated how much the lod score would increase at locus B by stratifying on the interacting locus to anticipate the question: Can stratification increase the evidence for linkage to the point that it becomes statistically significant when taking interaction into account? The increase in the lod score for the RR model was, maximally, 1.5 at the dataset size (50 families/dataset) that we examined. Those data sets that had a pre-pruning lod score value of 1.5, produced an average increase of at least 1 lod score unit after stratification, irrespective of the inheritance model. While as many as 20% of datasets showed false negatives (in which stratification caused a decrease in the lod score at locus B), we observed no false positive INT; no heterogeneity scenario produced any false positive results. This suggests that detecting a positive INT can be interpreted as strong evidence of gene-gene interaction, at least under the conditions of our tests.

In exploring the stratification approach using a disease-associated marker allele instead of a proven disease-causing allele, it was disappointing that the utility of the pruning approach was diminished when the LD between the associated allele and the disease allele (at locus A) needed be as high as 0.8–1.0. Such a requirement means that the marker allele either must *be* the disease allele or that the disease allele must almost always found in synteny with the marker allele in the population. It may be possible to increase the power to detect interaction by including other alleles at the marker locus besides the associated one.

In the work presented here, the correct model for the second disease locus (locus B) was always used in the analysis. Because lod score maximization will yield the correct mode of inheritance at a locus even under epistasis [12], the mode of inheritance for locus A is knowable. Interestingly, our simulations suggest that the INT will correctly indicate the presence of an interaction whatever the true inheritance model the second unknown interacting locus.

Our future simulations will explore the stratification procedure when linkage analyses are calculated under the wrong model in respect to the second unknown disease and examine the efficacy of using HLOD value instead of lod score value when heterogeneity exists. We will also take into consideration the scenario in which the disease is caused by three interacting loci.
